# Factors Associated with Primary Care Provider’s Job Satisfaction and Organizational Commitment in China: A Machine Learning-Based Random Forest Analysis

**DOI:** 10.3390/healthcare11101432

**Published:** 2023-05-15

**Authors:** Quan Wang, Siqi Liu, Yaqun Fu, Jiawei Zhang, Xia Wei, Zemeng Zhu, Ting Wang, Li Yang

**Affiliations:** 1 School of Public Health, Peking University, Beijing 100191, China; 2 Brown School, Washington University in St. Louis, St. Louis, MO 63130, USA; 3 Center of Health System and Policy, Institute of Medical Information & Library, Chinese Academy of Medical Sciences & Peking Union Medical College, Beijing 100020, China; 4 Department of Health Services Research and Policy, London School of Hygiene & Tropical Medicine, London WC1H 9SH, UK; 5 School of Basic Medicine Science, Shandong University, Jinan 250012, China; 6 School of Integrated Traditional Chinese and Western Medicine, Binzhou Medical University, Yantai 264003, China

**Keywords:** *job satisfaction*, *organizational commitment*, COVID-19, social restriction, primary care provider, China

## Abstract

The objective of the study is to explore the factors that influence the *job satisfaction* and *organizational commitment* of primary care providers in China, with a focus on the impact of the COVID-19 pandemic and the rescission of restriction policies. We utilized the 20-item Minnesota Satisfaction Questionnaire (MSQ) and the 25-item *organizational commitment* survey to assess *job satisfaction* and organizational commitment. In total, 435 valid responses were included in our analysis. The average scores for *job satisfaction* and *organizational commitment* were 80.6 and 90.8. After a two-step tuning process, we built random forest models by machine learning. The results show income change, working years, working years in the current institute, and age were the four most important features associated with *job satisfaction*, *organizational commitment,* and most of their dimensions. The number of professional fields engaged, gender, job status, and types of endowment insurance were least associated. During pandemic time, income-related factors remain a core concern for primary care providers, whereas job security may lose its importance. These findings suggest that financial bonuses may be an effective way to boost morale, and age-specific motivation plans may be necessary.

## 1. Introduction

The COVID-19 pandemic has had a significant impact on the world, with countries implementing measures to mitigate the spread of the virus (SARS-CoV-2) [1]. China was one of the first countries to experience the pandemic, and the Chinese government responded with strict restrictions on movement and social gatherings to control its spread [2], which had a considerable impact on the daily lives of citizens and healthcare workers, especially primary care providers [3,4].

China has a three-tiered healthcare system consisting of tertiary hospitals, secondary hospitals, and primary care institutes. Primary care institutes are a critical component of the Chinese healthcare system and offer a broad range of services, including health education, disease prevention, diagnosis, and treatment of common illnesses, maternal and child health services, and chronic disease management [5]. However, due to the lack of a gatekeeper function and imperfect payment methods, over 50% of outpatient admissions occur in hospitals rather than primary care institutes [6]. Consequently, primary care providers typically have more limited income and career prospects compared to their hospital counterparts [7].

Primary care providers have played a crucial role in responding to the COVID-19 pandemic. They have been on the front lines of the pandemic, providing care and treatment to those affected by the virus while also facing personal risks of infection [3]. The high workload, long working hours, and risk of infection have contributed to increased stress, anxiety, and burnout among all care providers [8,9], affecting their *job satisfaction* and *organizational commitment* [10]. Despite the significant progress in controlling the pandemic in China, primary care providers are still being impacted by COVID-19. The pandemic has created new challenges for the healthcare system and has highlighted the importance of *job satisfaction* and *organizational commitment* in maintaining the quality of care for patients. As the restrictions have been lifted, it is crucial to understand the factors that might influence the *organizational commitment* and *job satisfaction* of primary care providers, especially as they continue to manage the COVID-19 pandemic and deliver high-quality care for all patients.

*Job satisfaction* and *organizational commitment* are critical factors that could influence the performance of healthcare workers [11]. *Job satisfaction* is the level of contentment and fulfillment an individual derives from their job [11]. Intrinsic and extrinsic *job satisfaction* are two primary dimensions of *job satisfaction*. Intrinsic *job satisfaction* relates to the individual’s internal fulfillment derived from work, while extrinsic *job satisfaction* is related to external factors such as salary, work environment, and benefits [12]. *Organizational commitment* refers to the level of loyalty and dedication an individual has towards their organization [13], which has a significant predictive effect on employee turnover and retention [14,15]. *Organizational commitment* has five dimensions: affective, normative, ideal, economic, and choice commitment. *Affective commitment* refers to the emotional attachment an employee has towards the organization; *normative commitment* is related to the employee’s sense of responsibility and obligation towards the organization; *ideal commitment* relates to the employee’s values and beliefs aligning with the organization’s mission and vision; *economic commitment* is related to the financial benefits of staying with the organization; and *choice commitment* relates to the difficulty of leaving the organization [15].

Exploring the factors that influence *job satisfaction* and *organizational commitment* of primary care providers is critical for healthcare organizations to maintain a satisfied and committed workforce. During the last decade or so, there have been plenty of studies about this topic in China as well as in other countries all over the world [16,17,18]. Before the COVID-19 pandemic, age, gender, income, technical title, and workload were usually deemed to be several key indicators that were associated with *job satisfaction* [19,20]. The study about *organizational commitment* is relatively limited. In Rui’s study about whole mainland of China, *organizational commitment* is negatively associated with turnover intention [21]. We did not detect any study about *job satisfaction* or *organizational commitment* in the same region as this one, *except for* our previous paper, which suggested *normative commitment* can positively affect the other four dimensions of *organizational commitment* and *job satisfaction* for primary care providers [22]. However, few of them were conducted under a COVID-19 environment, and even fewer were focused on the rescission of restriction policies. This study can help identify the factors that most contribute to the *job satisfaction* and *organizational commitment* of primary care providers, providing insight for policymakers on how to improve the work environment, work-life balance, and motivation plans for healthcare workers.

## 2. Method

### 2.1. Study Settings and Design

This was a cross-sectional study that was conducted in District H of Jinan during 5–13 August 2020. District H, like other regions in China, implemented standard restriction policies during the COVID-19 pandemic, such as lockdowns, active case surveillance, and traffic restrictions. The lockdown policy was lifted on 17 February 2020, and most public facilities reopened on 1 April of the same year. District H has a healthcare system that is heavily focused on hospitals. While primary care is readily accessible, many residents still prefer hospitals and bypass primary care institutes, which is a common trend in other regions of China as well [5].

### 2.2. Participant and Data Collection

We employed a web-based survey approach to distribute questionnaires to all primary care providers (*n* = 989) in District H of Jinan. The survey was administered through Wenjuanxing, a webpage-based tool that allowed participants to complete the questionnaire using a computer or smartphone. Utilizing this approach enabled us to collect responses automatically and efficiently.

### 2.3. Instruments and Validity

To assess the *job satisfaction* of primary care providers, we used the Minnesota Satisfaction Questionnaire (MSQ) short form [23], which has been validated in Chinese and consists of 20 questions. Participants rated their level of satisfaction using a Likert scale, with scores ranging from one (very dissatisfied) to five (very satisfied). The Chinese version (in Mandarin) of the MSQ has been widely used in previous studies [24,25,26,27] and has demonstrated good internal consistency, with Cronbach’s alpha ranging from 0.88 to 0.93 [24,25,26]. The Chinese employee’s *Organizational commitment* questionnaire, originally developed by Ling in 2001 [15], was utilized to measure the *organizational commitment* of primary care providers in this study. However, recognizing the unique characteristics of healthcare workers, Gao made slight adjustments to the questionnaire and tested its validity [27]. The resulting scale had a Cronbach’s alpha range of 0.78 to 0.86 [27]. The scale consists of 25 Likert-type questions with scores ranging from 1 to 5, reflecting the five dimensions of *organizational commitment*: *affective commitment*, *normative commitment*, *ideal commitment*, *economic commitment*, and *choice commitment*. Thus, higher scores indicate greater *organizational commitment* among primary care providers. It is worth noting that this questionnaire has been extensively used in previous studies and has demonstrated good validity and reliability in the Chinese context.

### 2.4. Statistical Analysis

The demographic data of the participants was presented as frequencies and percentages to provide a clear understanding of the distribution of the sample. Descriptive statistics were utilized to explore the participants’ *job satisfaction* and *organizational commitment*, with measures such as frequency, percentage, mean, and standard deviation (SD) being calculated. These statistics allowed for a comprehensive understanding of the profile of the participants and provided a basis for the subsequent analysis.

We used machine learning-based random forest analysis to investigate the factors contributing to *job satisfaction* and *organizational commitment*. Random forest is an ensemble learning method that improves performance and reduces noise by combining the outputs of many parallel decision trees [28]. The dataset was split into training and testing sets, with the testing set comprising 20% of the data. In each round of analysis, we set *job satisfaction*, intrinsic and extrinsic *job satisfaction*, and *organizational commitment*, and its five dimensions as the target variables. We transformed all categorical features using the OneHotEncoder function, while numerical features were standardized using the StandardScaler function. Both functions were provided by the SciKit-Learn package. We conducted grid searches to obtain optimal hyperparameters for each algorithm, with five cross-validations and Root Mean Square Error (RMSE) and Mean Absolute Error (MAE) set as lost to determine the two best hyperparameters: n_estimators and max_features. We implemented a two-step tuning process, first conducting a grid search with n_estimators ranging from 1 to 100 with a 10-interval, followed by another grid search using n_estimators based on the previous results with a 1-interval, which applied a more granular grid search to fine-tune the number of estimators for optimal model performance. All statistical analyses were performed using Python 3.9 via Google Colab (Google Inc., Mountain View, CA, USA), which provided all team members with a uniform cloud-based development environment. It is of note that gradient-boosted trees could be a potential alternative for our study.

## 3. Results

A total of 528 primary care providers in District H participated in the survey, resulting in a response rate of 53.4%. After excluding incomplete or improperly filled responses, the final sample consisted of 435 valid responses.

### 3.1. Descriptive Results

The average age of respondents was 36 years, and the majority of participants were female (84.4%). Only 20.0% of respondents held a budgeted post, which represents a permanent employment relationship with a public institution, and the average length of employment in the current institution was 7.76 years, which is approximately half of their total years of experience (14.1 years). Approximately half of the respondents held a master’s degree (49.89%), followed by those with a bachelor’s degree (42.30%). The majority of participants were engaged in only one professional field (73.33%), with most being public health professionals or nurses. Participants spent more time on patient care-related work (72%) than on management work (20%). The average total scores for *job satisfaction* and *organizational commitment* were 80.60 and 90.75, respectively. The average intrinsic and extrinsic *job satisfaction* scores were 48.50 and 23.86. Regarding the dimensions of *organizational commitment*, the average scores for emotional commitment, *normative commitment*, *ideal commitment*, *economic commitment*, and *choice commitment* were 19.10, 19.54, 19.15, 18.54, and 14.42, respectively (refer to Table 1 for more information). We also detected possible *job satisfaction* and *organizational commitment* differences among different subgroups and corrections between different variables; see Supplementary Material Tables S1 and S2 for more information.

### 3.2. Random Forest Analysis

**Figure 1:**
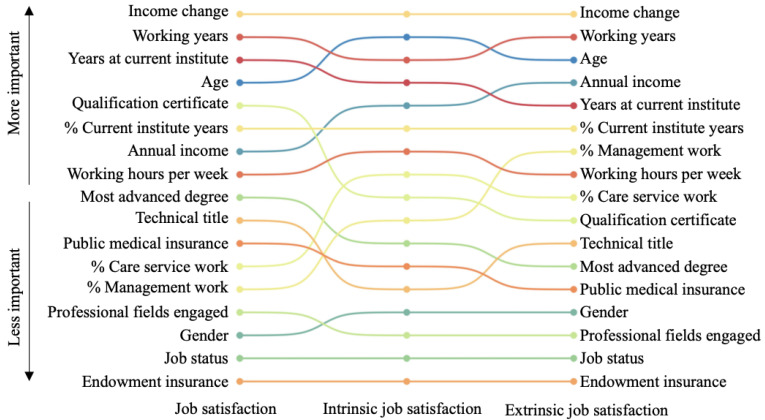
The rank of factors associated with *job satisfaction*.

Our analysis using machine learning-based random forests revealed that income change, working years, working years in the current institute, and age were the four most important features associated with *job satisfaction*, as well as intrinsic *job satisfaction*. On the other hand, annual incomes replaced working years in the current institute for extrinsic *job satisfaction*. Conversely, the number of professional fields engaged, gender, job status, and types of endowment insurance were least associated with *job satisfaction* and its two dimensions. Figure 1 illustrates the differences in importance scores among each feature; see Supplementary Material Table S3 for the full results.

**Figure 2:**
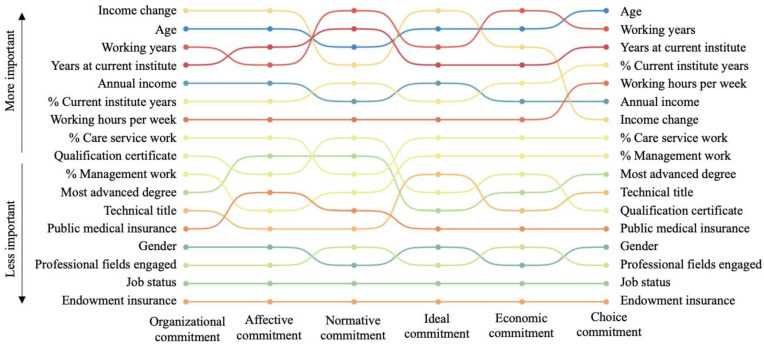
The rank of factors associated with organizational commitment.

The results of the analysis for *organizational commitment* and its five dimensions were similar to those for *job satisfaction*. Income change, working years, working years in the current institute, and age were the four most important features associated with *organizational commitment* and four of its dimensions (affective, normative, ideal, and economic commitment). In the case of choice commitment, income change was less important, and the proportion of working years at the current institute in the total working years took its place. Like the analysis for *job satisfaction*, the number of professional fields engaged, gender, job status, and types of endowment insurance were least associated with *organizational commitment*. Please refer to Figure 2 and Supplementary Material Table S4 for more information.

## 4. Discussion

Random forest is a robust machine learning classifier that exhibits strong predictive performance and can handle multicollinear relationships between a large group of variables [29]. It has been widely utilized for health outcome prediction [30], risk factor prioritization [31], and classification [32]. Although some studies have explored staffs’ working attitudes using random forest, their application to medical staff is still very limited. Our study demonstrates the effectiveness of random forest in the correlation analysis of working attitude, and we believe it could also be utilized for further studies on job burnout or other psychological conditions as an alternative to multiple linear regression. Our study contributes to the policy field by demonstrating the effectiveness of random forest in exploring the important associated factors of *job satisfaction* and *organizational commitment*. To the best of our knowledge, the application of random forest in this area is very limited, making our study novel and unique. Moreover, the use of random forest as an alternative to ANOVA and multiple linear regression is an important contribution to the methodological literature. ANOVA assumes equal variances and independence of observations, which can be violated in practice and lead to biased results. Multiple linear regression assumes linearity and independence between the predictors, which can also be unrealistic in many settings. Random forest is a non-parametric method that does not make any assumptions about the underlying data distribution or the functional form of the relationships between the predictors and the outcome. This makes it more flexible and robust to violations of the underlying assumptions than traditional parametric methods.

Due to China’s hospital-centered healthcare system, research on primary care providers’ *job satisfaction* and *organizational commitment* has been limited compared to hospital doctors. Furthermore, studies on the impact of COVID-19 on these factors are rare. This study is the first to examine the factors that influence *job satisfaction* and *organizational commitment* among primary care providers in China after the lifting of restriction policies. The results indicate that income change, working years, working years in the current institute, and age were the four most important factors associated with *job satisfaction*, *organizational commitment*, and their subdimensions. On the other hand, the number of professional fields engaged, gender, job status, and types of endowment insurance were the least associated factors.

Income-related factors have consistently been shown to be significant predictors of *job satisfaction* and *organizational commitment* among primary care providers in China, both before and after the COVID-19 pandemic [33,34]. However, in this study, income change was found to be a more important factor than income level itself, although income level still ranked near the top. One possible explanation is the impact of COVID-19 on the economy, which has negatively affected many people’s incomes and led to increased job insecurity. In contrast, primary care providers have enjoyed relatively stable incomes and financial expectations, even receiving some government subsidies. Thus, the level of income may have been less influential in comparison. Another possible explanation is that income change may reflect factors beyond just the level of income, such as career advancement, promotions, or increased responsibilities, which are more closely linked to *job satisfaction* and *organizational commitment* than just the level of income. The Equity Theory by Adams suggests that employees compare their inputs and outputs to those of others, and when there is a perceived imbalance, they will take action to restore equity [35]. In this study, the income change factor may have reflected a sense of fairness or equity, as primary care providers saw their incomes increase or stabilize while others experienced decreases.

This study also revealed that working years and working years in the current institute were important factors associated with these outcomes. Longer working years often indicate a higher level of experience and expertise, which can contribute to greater *job satisfaction* and commitment to the organization. As primary care providers gain more experience, they may feel more confident in their ability to provide quality care and may also enjoy greater autonomy in their work. In addition, primary care providers who have worked at the same institute for a longer period of time may have developed strong relationships with colleagues and patients, which could increase their sense of belonging and commitment to the organization.

We found that the number of professional fields engaged, gender, job status, and types of endowment insurance were least associated with *job satisfaction* and *organizational commitment* among primary care providers in China. Prior research has suggested that there may be differences in *job satisfaction* and *organizational commitment* between primary care providers of different genders [36,37]. We believe that the high pressure and workload caused by the COVID-19 pandemic may have led all primary care providers to work harder, and thus, the differences between genders were less pronounced. It is interesting to note that job status, which has traditionally been a core concern of employment, did not seem to have a significant influence on *job satisfaction* and *organizational commitment* among primary care providers in our study. We speculate that the pandemic may have caused primary care providers to realize the value of their professional skills, and they were less afraid of changing working institutions as the need for their skillset was in high demand. In fact, the scores for *choice commitment* were the lowest dimension of *organizational commitment*, which suggests that primary care providers may not highly value their current job and may be more open to exploring other opportunities.

These findings have important implications for policymakers seeking to maximize *job satisfaction* and *organizational commitment* among primary care providers. Firstly, income-related factors remain a core concern for primary care providers, and financial bonuses may be an effective way to boost morale. Secondly, age-specific motivation plans may be necessary given the partial impact of COVID-19 on older primary care providers. Additionally, our findings suggest that job security may be losing popularity among primary care providers, making income and a sense of social duty even more important.

## 5. Limitation

However, like any method, random forest also has its limitations. One potential drawback is that it can be computationally intensive, especially for large datasets with many features. Another limitation is that it may be prone to overfitting if not properly tuned, which can lead to reduced prediction performance on new data. To address these limitations, we carefully selected our hyperparameters, including the maximum number of features and number of estimators, to optimize the model’s performance.

Another limitation is that our sample size was relatively small, which may limit the generalizability of our findings. Additionally, our study was conducted in a district of Jinan, which may limit the generalizability of our findings to other healthcare settings.

## 6. Conclusions

Income-related factors remain a core concern for primary care providers, whereas job security may lose its importance. Age and working years should also be given full consideration when policymakers try to maximize *job satisfaction* and *organizational commitment* among primary care providers.

## Figures and Tables

**Table 1:** Characteristics of included participants (*n* = 435).

Discrete Variables	Number (%)
Gender	
Men	68 (15.63)
Women	367 (84.37)
Job status	
Budgeted post	87 (20.00)
Temporary contract labor	348 (80.00)
Professional qualification certificate	
No qualification certificate has been obtained yet	29 (6.67)
Qualification certificate of medical practitioner	100 (22.99)
Qualification certificate of licensed assistant physician	21 (4.83)
Village doctor’s practice certificate	3 (0.69)
Other health technology qualification certificates	282 (64.83)
Technical title	
Senior	1 (0.23)
Vice senior	18 (4.14)
Intermediate	99 (22.76)
Medical practitioner or occupation (assistant doctor)	89 (20.46)
Others	228 (52.41)
Most advanced degree	
Doctor	13 (2.99)
Master	217 (49.89)
Bachelor	184 (42.30)
Junior college	21 (4.83)
Senior high school and below	0 (0)
Professional field(s) engaged	
Medical treatment (traditional Chinese medicine not included)	73 (17.87)
Traditional Chinese medicine	49 (11.26)
Public health services	174 (40.00)
Nursing	165 (37.93)
Others	110 (25.29)
Number of professional field(s) engaged	
1	319 (73.33)
2	98 (22.53)
3	17 (3.91)
5	1 (0.23)
Income change *	
A significant improvement	17 (3.91)
An improvement	193 (44.37)
No change	188 (43.22)
A decline	30 (6.90)
A significant decline	7 (1.61)
Public medical insurance	
Medical insurance for urban and rural residents	37 (8.51)
Medical insurance for urban residents	311 (71.49)
No public medical insurance	87 (20.00)
Endowment insurance	
Basic endowment insurance for urban workers	412 (94.71)
Basic endowment insurance for urban and rural residents	12 (2.76)
No endowment insurance	11 (2.53)
**Continuous variables:**	**Mean (SE)**
Age (years)	36.20 ± 8.72
Working years (years)	14.12 ± 8.85
Working years in the current institute (years)	7.76 ± 8.20
Proportion of time occupied by management work (%)	20.45 ± 30.57
Proportion of time occupied by care service work (%)	72.36 ± 34.79
Annual income from medical practice in the 2019 (RMB)	48,400 ± 25,200
Working hours per week	41.73 ± 10.61
Job satisfaction	80.60 ± 13.80
Intrinsic job satisfaction	48.50 ± 8.21
Extrinsic job satisfaction	23.86 ± 4.64
Organizational commitment	90.75 ± 16.74
Affective commitment	19.10 ± 3.66
Normative commitment	19.54 ± 3.45
Ideal commitment	19.15± 4.08
Economic commitment	18.54 ± 3.93
Choice commitment	14.42 ± 4.59

*: Self-reported income from medical practice in 2019, compared with 2018.

## Data Availability

The datasets generated and/or analyzed during the current study are not publicly available due to further research but are available from the corresponding author on reasonable request.

## Supplementary Materials

The following supporting information can be downloaded at: https://www.mdpi.com/article/10.3390/healthcare11101432/s1, Table S1: Characteristics of included participants; Table S2: Correction of variables; Table S3: The results of tune process and feature importance of job satisfaction; Table S4: The results of tune process and feature importance of organizational commitment.
