# Supplementary material for: Factors Associated with Primary Care Provider’s Job Satisfaction and Organizational Commitment in China: A Machine Learning-Based Random Forest Analysis
Source: Healthcare (Basel). 2023 May 15;11(10):1432. doi: 10.3390/healthcare11101432 (PMC10218293; doi:10.3390/healthcare11101432)
Supplement: Supplementary file 1 [file healthcare-11-01432-s001.zip › healthcare-2301993-supplementary.pdf]

Supplementary Materials

Table S1 Characteristics of included participants (N=435)

**Discrete variables**	**Number (%)**	p_j[†]	p_o[‡]
Gender		0.234	0.796
Men	68(15.63)		
Women	367(84.37)		
Age		0.081	0.006
21~29	100(23.00)		
31~39	192(44.13)		
41~49	111(25.52)		
51~59	28(6.44)		
60~	4(0.92)		
Working years		0.681	0.452
0~5	67(15.63)		
6~10	115(26.44)		
11~15	106(24.37)		
16~20	52(11.95)		
21~25	38(8.42)		
26~30	36(8.28)		
31~26	12(2.76)		
36~	7(1.61)		
Job status		0.499	0.002

Budgeted post	87(20.00)		
Temporary contract labor	348(80.00)		
Professional qualification certificate		0.647	0.937
No qualification certificate has been obtained yet	29(6.67)		
Qualification certificate of medical practitioner	100(22.99)		
Qualification certificate of licensed assistant physician	21(4.83)		
Village doctor's practice certificate	3(0.69)		
Other health technology qualification certificates	282(64.83)		
Technical title:		0.720	0.597
Senior	1(0.23)		
Vice senior	18(4.14)		
Intermediate	99 (22.76)		
Medical practitioner or occupation (assistant doctor)	89(20.46)		
Others	228(52.41)		
Most advanced degree		0.613	0.407
Doctor	13(2.99)		
Master	217(49.89)		
Bachelor	184(42.30)		
Junior college	21(4.83)		
Senior high school and below	0(0)		
Number of professional field(s) engaged		0.474	0.165
1	319(73.33)		
2	98(22.53)		
3	17(3.91)		
5	1(0.23)		
Income change*		<0.001	<0.001

A significant improvement	17(3.91)		
An improvement	193(44.37)		
No change	188(43.22)		
A decline	30(6.90)		
A significant decline	7(1.61)		
Public medical insurance		0.159	0.650
Medical insurance for urban and rural residents	37(8.51)		
Medical insurance for urban residents	311(71.49)		
No public medical insurance	87(20.00)		
Endowment insurance		0.949	0.748
Basic endowment insurance for urban workers	412(94.71)		
Basic endowment insurance for urban and rural residents	12(2.76)		
No endowment insurance	11(2.53)		

*: Self-reported income from medical practice in 2019, compared with 2018

†: The *p* value of t test or ANOVA for job satisfaction among different subgroups

‡: The *p* value of t test or ANOVA for organizational commitment among different subgroups

Table S2 Correction of variables

		Age	Working years	Years at current institute	% Current institute years	Care service work	Annual income	Working hours per week	Organizational commitment	Job satisfaction
Age	Pearson correlation coefficient									
Working years	Pearson correlation coefficient	0.927**								
Years at current institute	Pearson correlation coefficient	0.576**	0.635**							
% Current institute years	Pearson correlation coefficient	-0.113*	-0.124**	0.501**						
Care service work	Pearson correlation coefficient	-0.129**	-0.103*	0.015	0.084					
Annual income	Pearson correlation coefficient	0.325**	0.311**	0.357**	0.103*	-0.026				
Working hours per week	Pearson correlation coefficient	0.050	0.055	0.081	0.029	0.071	0.058			
Organizational commitment	Pearson correlation coefficient	0.166**	0.162**	0.138**	0.103*	-0.020	0.081	-0.030		
Job satisfaction	Pearson correlation coefficient	0.095*	0.090	0.026	0.014	0.020	0.075	-0.031	0.763**	

**: correlation is significant at the 0.01 level (two-tailed)

*: correlation is significant at the 0.05 level (two-tailed)

Table S3 The results of tune process and feature importance of job satisfaction

Job satisfaction		Intrinsic job satisfaction		Extrinsic job satisfaction	
**Tuning (first step)**		**Tuning (first step)**		**Tuning (first step)**	
Max features	5	Max features	8	Max features	9
N estimators	20	N estimators	90	N estimators	30
**Tuning (second step)**		**Tuning (second step)**		**Tuning (second step)**	
Max features	3	Max features	14	Max features	7
N estimators	29	N estimators	36	N estimators	13
RMSE	13.0396	RMSE	8.4158	RMSE	4.5427
MAE	9.9473	MAE	6.1076	MAE	3.4987
**Features**	**Importance**	**Features**	**Importance**	**Features**	**Importance**
Self-reported income from medical practice in 2019, compared with 2018	0.1725	Self-reported income from medical practice in 2019, compared with 2018	0.1434	Self-reported income from medical practice in 2019, compared with 2018	0.1373
Working years	0.1161	Age	0.1021	Working years	0.0974
Working years at the current institute	0.1107	Working years	0.0979	Age	0.0907
Age	0.109	Working years at the current institute	0.0884	Annual income from medical practice in the 2019	0.0892
Professional qualification certificate	0.0984	Annual income from medical practice in the 2019	0.0830	Working years at the current institute	0.0742
Proportion of working years at the current institute in whole working years	0.0791	Proportion of working years at the current institute in whole working years	0.0794	Proportion of working years at the current institute in whole working years	0.0734
Annual income from medical	0.0754	Working hours per week	0.0617	Proportion of time occupied by	0.0673

practice in the 2019				management work	
Working hours per week	0.0736	Proportion of time occupied by care service work	0.0588	Working hours per week	0.0667
Most advanced degree	0.068	Professional qualification certificate	0.0437	Proportion of time occupied by care service work	0.0617
Technical title	0.0501	Proportion of time occupied by management work	0.0433	Professional qualification certificate	0.0495
Types of public medical insurance	0.0485	Most advanced degree	0.0427	Technical title	0.0406
Proportion of time occupied by care service work	0.0472	Types of public medical insurance	0.0401	Most advanced degree	0.0391
Proportion of time occupied by management work	0.0406	Technical title	0.0323	Types of public medical insurance	0.0339
Number of professional fields engaged	0.0272	Gender	0.0292	Gender	0.0280
Gender	0.0254	Number of professional fields engaged	0.0269	Number of professional fields engaged	0.0246
Job status	0.0134	Job status	0.0166	Job status	0.0143
Types of endowment insurance	0.0129	Types of endowment insurance	0.0106	Types of endowment insurance	0.0122

Table S4 The results of tune process and feature importance of organizational commitment

Organizational commitment		Affective commitment		Normative commitment	
**Tuning (first step)**		**Tuning (first step)**		**Tuning (first step)**	
Max features	29	Max features	8	Max features	8
N estimators	60	N estimators	40	N estimators	40
**Tuning (second step)**		**Tuning (second step)**		**Tuning (second step)**	
Max features	14	Max features	3	Max features	7
N estimators	52	N estimators	43	N estimators	46
RMSE	15.5829	RMSE	3.6726	RMSE	3.1386
MAE	12.5376	MAE	2.9254	MAE	2.4008
**Features**	**Importance**	**Features**	**Importance**	**Features**	**Importance**
Self-reported income from medical practice in 2019, compared with 2018	0.1475	Self-reported income from medical practice in 2019, compared with 2018	0.1045	Working years	0.1161
Age	0.1181	Age	0.1033	Working years at the current institute	0.1107
Working years	0.0905	Working years at the current institute	0.0989	Age	0.1090
Working years at the current institute	0.0891	Working years	0.0926	Self-reported income from medical practice in 2019, compared with 2018	0.1036
Annual income from medical practice in the 2019	0.0888	Annual income from medical practice in the 2019	0.0850	Proportion of working years at the current institute in whole working years	0.0791
Proportion of working years at the current institute in whole working	0.0763	Proportion of working years at the current institute in whole working	0.0845	Annual income from medical practice in the 2019	0.0754

years		years			
Working hours per week	0.0750	Working hours per week	0.0679	Working hours per week	0.0736
Proportion of time occupied by care service work	0.0483	Proportion of time occupied by care service work	0.0550	Professional qualification certificate	0.0480
Professional qualification certificate	0.0442	Most advanced degree	0.0501	Most advanced degree	0.0476
Proportion of time occupied by management work	0.0411	Professional qualification certificate	0.0481	Proportion of time occupied by care service work	0.0472
Most advanced degree	0.0411	Types of public medical insurance	0.0398	Proportion of time occupied by management work	0.0406
Technical title	0.0337	Proportion of time occupied by management work	0.0385	Types of public medical insurance	0.0349
Types of public medical insurance	0.0328	Technical title	0.0380	Technical title	0.0339
Gender	0.0320	Gender	0.0326	Number of professional fields engaged	0.0272
Number of professional fields engaged	0.0188	Number of professional fields engaged	0.0252	Gender	0.0269
Job status	0.0159	Job status	0.0194	Job status	0.0134
Types of endowment insurance	0.0068	Types of endowment insurance	0.0165	Types of endowment insurance	0.0129

Table S4 The results of tune process and feature importance of organizational commitment (continued)

Ideal commitment		Economic commitment		Choice commitment	
**Tuning (first step)**		**Tuning (first step)**		**Tuning (first step)**	
Max features	10	Max features	6	Max features	16

N estimators	80	N estimators	80	N estimators	90
**Tuning (second step)**		**Tuning (second step)**		**Tuning (second step)**	
Max features	4	Max features	6	Max features	7
N estimators	80	N estimators	80	N estimators	98
RMSE	4.1105	RMSE	3.9090	RMSE	4.4049
MAE	3.3080	MAE	3.1820	MAE	3.5431
**Features**	**Importance**	**Features**	**Importance**	**Features**	**Importance**
Self-reported income from medical practice in 2019, compared with 2018	0.1484	Working years	0.1166	Age	0.1072
Age	0.0966	Age	0.1089	Working years	0.0981
Working years	0.0934	Self-reported income from medical practice in 2019, compared with 2018	0.0998	Working years at the current institute	0.0947
Working years at the current institute	0.0822	Working years at the current institute	0.0886	Proportion of working years at the current institute in whole working years	0.0892
Annual income from medical practice in the 2019	0.0746	Proportion of working years at the current institute in whole working years	0.0849	Working hours per week	0.0856
Proportion of working years at the current institute in whole working years	0.0739	Annual income from medical practice in the 2019	0.0846	Annual income from medical practice in the 2019	0.0776
Working hours per week	0.0600	Working hours per week	0.0644	Self-reported income from medical practice in 2019, compared with 2018	0.0756

Proportion of time occupied by care service work	0.0532	Proportion of time occupied by care service work	0.0510	Proportion of time occupied by care service work	0.0584
Proportion of time occupied by management work	0.0478	Proportion of time occupied by management work	0.0456	Proportion of time occupied by management work	0.0523
Technical title	0.0474	Professional qualification certificate	0.0448	Most advanced degree	0.0519
Professional qualification certificate	0.0463	Most advanced degree	0.0428	Technical title	0.0493
Most advanced degree	0.0463	Technical title	0.0423	Professional qualification certificate	0.0422
Types of public medical insurance	0.0401	Types of public medical insurance	0.0410	Types of public medical insurance	0.0401
Gender	0.0301	Number of professional fields engaged	0.0249	Gender	0.0238
Number of professional fields engaged	0.0284	Gender	0.0248	Number of professional fields engaged	0.0205
Job status	0.0171	Job status	0.0187	Job status	0.0201
Types of endowment insurance	0.0143	Types of endowment insurance	0.0163	Types of endowment insurance	0.0133
